# Wnt Signaling in the Regulation of Immune Cell and Cancer Therapeutics

**DOI:** 10.3390/cells8111380

**Published:** 2019-11-03

**Authors:** Muhammad Haseeb, Rameez Hassan Pirzada, Qurat Ul Ain, Sangdun Choi

**Affiliations:** Department of Molecular Science and Technology, Ajou University, Suwon 16499, Korea; haseeb3389@hotmail.com (M.H.); rameez_hassan99@yahoo.com (R.H.P.); ainne.w@gmail.com (Q.U.A.)

**Keywords:** Wnt signaling, immune cell regulation, cancer, therapeutic target, inhibitor

## Abstract

Wnt signaling is one of the important pathways to play a major role in various biological processes, such as embryonic stem-cell development, tissue regeneration, cell differentiation, and immune cell regulation. Recent studies suggest that Wnt signaling performs an essential function in immune cell modulation and counteracts various disorders. Nonetheless, the emerging role and mechanism of action of this signaling cascade in immune cell regulation, as well as its involvement in various cancers, remain debatable. The Wnt signaling in immune cells is very diverse, e.g., the tolerogenic role of dendritic cells, the development of natural killer cells, thymopoiesis of T cells, B-cell-driven initiation of T-cells, and macrophage actions in tissue repair, regeneration, and fibrosis. The purpose of this review is to highlight the current therapeutic targets in (and the prospects of) Wnt signaling, as well as the potential suitability of available modulators for the development of cancer immunotherapies. Although there are several Wnt inhibitors relevant to cancer, it would be worthwhile to extend this approach to immune cells.

## 1. Introduction

Wnt signaling performs a wide variety of essential tasks in the human body by regulating cell differentiation, proliferation, embryonic growth, stem cell development, immune cell functions, and tissue repair and regeneration [1,2]. Various communication mechanisms such as chemical signals exist between the Wnt protein and a receptor called Frizzled (FZD), to execute these tasks in a coordinated manner. In mammals, there are 19 distinct Wnt-type ligands (hereafter “Wnt ligands”), which bind to various receptors including 10 FZD proteins, and numerous co-receptors such as lipoprotein receptor–related proteins (LRP5 and LRP6). The Nusse laboratory at Stanford University has done tremendous work and gathered a large amount of information on Wnt signaling (see “The Wnt homepage” www.stanford.edu/group/nusselab/cgi-bin/wnt/). Wnt signaling is classified into canonical and noncanonical pathways: (1) the canonical one involves cadherin-associated protein β (β-catenin), T-cell factor (TCF), and lymphocyte enhancer-binding factor (LEF); and (2) the noncanonical pathway, includes the planar cell polarity (PCP) pathway and Wnt calcium pathway (Wnt–Ca^2+^) [3]. Dysregulation of the Wnt signaling cascade and aberrant expression of Wnt ligands causes several disorders such as autoimmune diseases, osteoarthritis, asthma, allergy, and cancer [2,4]. Recently, immunologists reported in a number of studies that Wnt signaling and Wnt ligands play an essential and considerable role in the regulation of immune cells. Therefore, a detailed study is needed to understand the mechanisms of the Wnt signaling pathway and the impact of Wnt ligands on immune cell modulation and various diseases. Here, we review the mechanisms of Wnt signaling and the effects of Wnt ligands on immune cell modulation. First, we briefly explain the activation mechanisms of the canonical and noncanonical Wnt signaling pathways. Then, we discuss the involvement of Wnt signaling and recent findings of Wnt ligands and related proteins in the regulation of such immune cells as dendritic cells (DCs), natural killer (NK) cells, T cells, macrophages, and B cells. Next, we address the participation of Wnt signaling and the respective ligands in breast cancer, leukemia, gastrointestinal cancers (GCs), and brain cancers. Lastly, we highlight the current therapeutic targets in Wnt signaling and the possible suitability of the available modulators for the development of cancer immunotherapies.

## 2. Molecular Players in Canonical and Noncanonical Wnt Pathways

### 2.1. Wnt–β-Catenin Signaling

**Figure 1:**
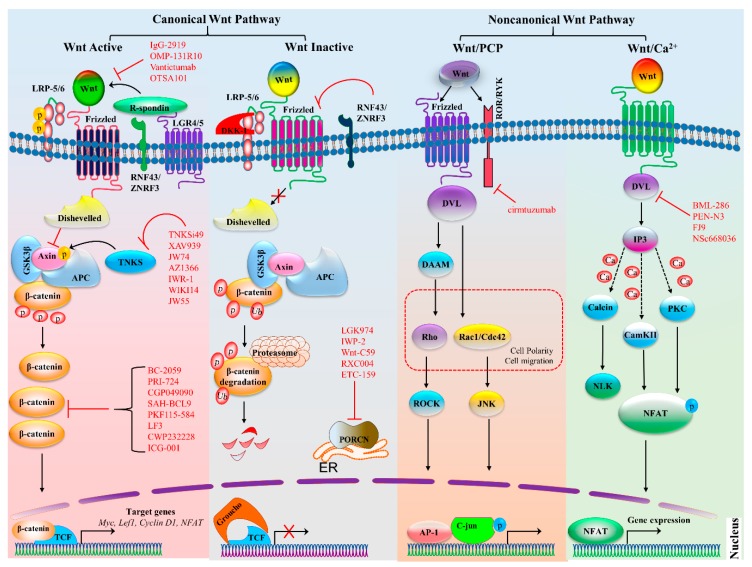
Canonical and noncanonical Wnt signaling pathways. The canonical Wnt pathway is activated by the binding of a Wnt ligands to a frizzled (FZD) family receptor and co-receptor LRP5 or LRP6, which recruits disheveled (Dvl), consequently inactivating the destruction complex composed of APC, GSK3β, and axin. This inactivation prevents β-catenin from proteasomal degradation and allows for the accumulation of β-catenin, which then enters the nucleus. There, it binds to transcription factor TCF or LEF and initiates the transcription of target genes. Tankyrases (TNKSs) also promote signaling by targeting axin for degradation. Moreover, when R-spondin binds to LGR4 or LGR5, RNF43, and ZNRF3, it is not capable of targeting FZD family receptors for degradation and enhances Wnt signaling. There are various inhibitors of the Wnt signaling pathway, particularly those targeting Wnt ligands, Dvl, TNKS, β-catenin, and PORCN, which are highlighted in red; bars indicate the inhibitory effect. In the absence of Wnt ligands, the destruction complex becomes active and starts the proteasomal degradation of β-catenin. Proteins RNF43 and ZNRF3 also inhibit the binding of FZD and target it for degradation. The noncanonical Wnt–PCP pathway is triggered by Wnt ligands that increase the heterodimerization of a receptor-like tyrosine kinase (RYK) and tyrosine kinase–like orphan receptor (ROR). The binding to the receptor activates the Dvl protein and downstream signaling, for instance, DAAM activates GTPases Rho and ROCK, whereas the activation of c-Jun N-terminal kinase (JNK) by Rac is independent of DAAM. They collectively regulate cell polarity and migration and have also been implicated in cancer. The Wnt–Ca^2+^ pathway is activated by ligand Wnt, which raises the intracellular Ca^2+^ levels and generates inositol 1,4,5-triphosphate-3 (IP3). The Ca^2+^ levels increase and switch on downstream Ca^2+^-dependent enzymes such as calmodulin-dependent protein kinase (CaMKII), calcineurin, and protein kinase C (PKC). As a consequence, CaMKII and PKC phosphorylate nuclear factor of activated T cells (NFAT) and activate the expression of target genes. Protein symbols and abbreviations: APC, adenomatous polyposis coli protein; AP-1, activator protein 1; DKK1, dickkopf related protein 1; ER, endoplasmic reticulum; GSK3β, glycogen synthase kinase 3β; LEF1, lymphoid enhancer-binding factor 1; LRP, lipoprotein receptor-related protein; LGR4/5, Leucine-rich-repeat–containing G protein–coupled receptor 4 or 5; NLK, Nemo like kinase; RNF43, Ring finger protein 43; and ZNRF3, zinc ring finger 3.

In Wnt–β-catenin signaling, activation of the cascade is triggered by the binding of a Wnt ligand (Wnt1, Wnt2, Wnt3, Wnt3a, Wnt7a, Wnt7b, Wnt8a, Wnt8b, Wnt10b, or Wnt16) to its respective FZD family receptor (ten such receptors in humans and mice) and low-density co-receptor LRP5 or LRP-6 (Figure 1) [5,6,7]. The activation of the Wnt receptor complex triggers downstream signaling, which causes the accumulation of β-catenin in the cytoplasm and its eventual translocation into the nucleus, where it can interact with transcription factors TCF and LEF and activate Wnt target genes cyclin D1 (*CCND1*), AXIN2, the *Myc* proto-oncogene, and dickkopf 1 (*DKK1*) [8]. When Wnt signaling is inactive, β-catenin levels are kept low by a destruction complex. This complex is composed of protein kinases, including casein kinase 1 (CK1), glycogen synthase kinase 3 β (GSK3β), adenomatous polyposis coli (APC), and axin. The activation of the Wnt–β-catenin signaling pathway is involved in many cellular functions: cell cycle regulation, cell proliferation, apoptosis, stem cell development, differentiation of progenitor cells, and immune cell regulation [1,2]. Recently, it was reported that canonical Wnt–TCF signaling can regulate immune cell–mediated responses of T cells and DCs and stabilizes immunity [9]. Consequently, the aberrant activation of this cascade causes various immune disorders and cancers.

### 2.2. Noncanonical Wnt Signaling

In the noncanonical Wnt pathway, signaling is activated upon binding of a Wnt5a class ligand (Wnt4, Wnt5a, Wnt5b, Wnt6, or Wnt11). The noncanonical Wnt signaling pathway is classified into 2 pathways: the PCP pathway and Wnt–Ca^2+^ pathway (Figure 1). The PCP pathway takes part in the regulation of cellular polarization, adhesion, stem cell maintenance, embryonic development, and cell invasion and migration [10]. Molecular factors of the Wnt signaling pathway, typically Wnt4, Wnt5a, Wnt5b, and Wnt11, bind to receptors of the FZD family, receptor-like tyrosine kinase (RYK), receptor tyrosine kinase–like orphan receptor 1 or 2 (ROR1 or ROR2), and protein tyrosine kinase 7 (PTK7), which trigger the noncanonical PCP pathway. There are several other important components of the Wnt–PCP pathway including Dvl1-3, prickle-like proteins 1–4 (PRICKLE1–4), Vang-like proteins 1 and 2 (VANGL1/2), cadherin EGF LAG seven-pass G-type receptors 1–3 (CELSR1–3), and small G proteins Rho and Ras-related C3 botulinum toxin substrate 1 (Rac1) [11]. In the PCP pathway, signals are transformed to actin cytoskeletal movements through Rho and Rac, and then, Rho-associated kinase activates Jun N-terminal kinase (JNK)-dependent transcription. Recently, it was reported that receptor RYK might regulate the development of NK cells, and the role of RYK in hematopoiesis was proposed [12]. Moreover, noncanonical Wnt signaling via receptor tyrosine kinases (RTKs), such as ROR1, ROR2, and RYK, activates phosphatidylinositol-3 kinase–AKT (PI3K–AKT) signaling and is involved in numerous cancers including breast cancer, GCs, leukemia, and brain cancer [13,14]. 

In the noncanonical Wnt–Ca^2+^ pathway, ligand Wnt5a interacts with receptor FZD along with co-receptor ROR1 or ROR2, causing a release of Ca^2+^ from the endoplasmic reticulum and activating phospholipase C (PLC) through G protein and SEC14-like protein 2 (SEC14L2), resulting in the formation of 1,2-diacylglycerol (DAG) and inositol 1,4,5-triphosphate (IP3) [13,15]. Previously, it has been revealed that Wnt ligands activate several downstream Ca^2+^-dependent enzymes such as calcineurin, protein kinase C (PKC), and calmodulin-dependent kinase II (CaMKII) [16,17]. Moreover, calcineurin, PKC, and CaMKII participate in various cellular phenomena including cell differentiation, migration, and adhesion through the regulation of transcription factors myocyte enhancer factor 2 (MEF2) and nuclear factor of activated T cells (NFAT) [18].

Noncanonical Wnt ligands substantially participate in the inhibition of canonical signaling; calcineurin and CaMKII stimulation triggers Nemo-like kinase (NLK) and inhibits Wnt–β-catenin signaling [19]. It has been stated that the Wnt–Ca^2+^ signaling pathway is strongly associated with tumorigenesis and cancer progression. Several studies show that ligand Wnt5a often activates the Wnt–Ca^2+^ pathway in cancer cells and higher expression of Wnt5a suppresses breast and colorectal cancer (CRC) [20,21]. Collectively, the detailed mechanisms of action of canonical and noncanonical Wnt ligands, and their signaling cascades in immune cells with respect to diseases and treatments, are still debated and require research for the subsequent development of therapeutics.

## 3. Wnt Signaling in Immune Cell Regulation

**Figure 2:**
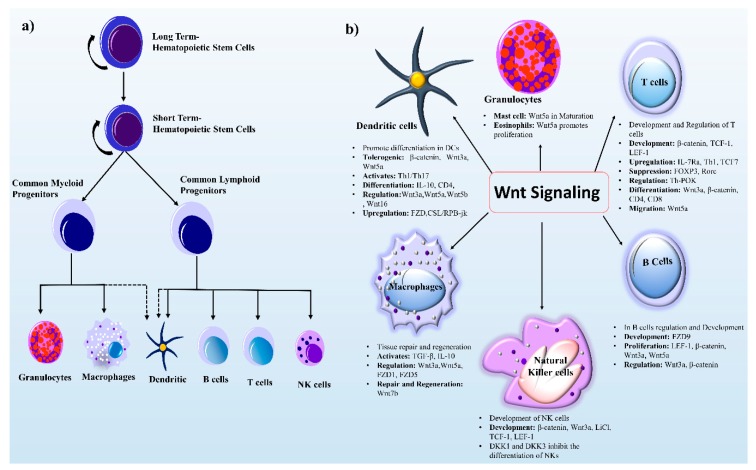
HSC development and Wnt signaling components in immune cell regulation. (**a**) Differentiated blood cells are generated from self-renewing LT-HSCs, which are capable of differentiation and self-renewal. When LT-HSCs differentiate, they form ST-HSCs with a limited self-renewal capability. The ST-HSCs next produce the multipotent non self-renewing common myeloid linage (granulocytes and macrophages) and common lymphoid lineage (B cells, T cells, and NK cells). The dashed lines show partial progenitor connections. (**b**) Wnt signaling components play role in myeloid and lymphoid lineage cells. Wnt ligands (Wnt3a, Wnt5a, Wnt5b, and Wnt16) and receptors (FZD1 and FZD5) take part in the regulation of immune cells. The major role of Wnt signaling in B cells, T cells, and NK cells is development. By contrast, in macrophages, this signaling governs tissue repair and regeneration. Wnt signaling and its components perform different tasks in immune cells such as activation, proliferation, migration, tolerogenesis, and up- and down-regulation of genes as shown in text boxes. Protein symbols and abbreviations: CD4, cluster of differentiation 4; DCs, dendritic cells; DKK, dickkopf-related protein; FZD, frizzled; FOXP3, forkhead box P3; IL-10, interleukin 10; IL7Ra, interleukin 7 receptor α; LEF1, lymphoid enhancer-binding factor 1; LiC1, ligand-gated ion channel; NKs, natural Killer cells; RORC, related orphan receptor C; TH1, T helper 1 cell; TGFβ, transforming growth factor β; Th-POK, a zinc finger protein.

The development and regulation of immune cells originate from hematopoietic stem cells (HSCs). The latter can retain self-renewal enabling differentiation and regeneration to produce mature blood cells is critical to survival [22]. During development, murine embryonic hematopoiesis takes place at different anatomical sites (extraembryonic yolk sac, placenta, and aorta-gonad-mesonephros (AGS)). These HSCs then move into and seed different organs; normal adult hematopoiesis occurs in bone marrow and follows a series of developmental steps (Figure 2a). Furthermore, the response of HSCs can be divided into two phases: (a) Long-term HSCs (LT-HSCs) possess the capacity for both differentiation and self-renewal for a long time, (b) and short-term HSCs (ST-HSCs) can perform restoration for a limited time. These ST-HSCs give rise to multipotent progenitor cells, myeloid lineage cells (macrophages and granulocytes), and lymphoid progenitor cells (NK cells, dendritic cells, B cells, and T cells), while DCs have partial lineage connections. In this review, we mainly focus on the role of Wnt signaling in lymphoid and myeloid progenitor cells.

In this context, Wnt signaling is a key player and a vital part of immune cell modulation, development, activation, regeneration, and downregulation (Figure 2b). The involvement of Wnt signaling in the immune system was initially reported in relation to T-cell development in the thymus [3]. The Wnt–β-catenin signaling pathway is widely studied because it helps to direct immune cell infiltration and is looming large as a new putative target for molecular therapeutics of cancer [23].

### 3.1. Wnt Signaling in Lymphoid-Originated Immune Cells

#### 3.1.1. Wnt Signaling in DCs

DCs are antigen-presenting cells of the mammalian immune system that regulate an adaptive immune response. DCs are essential for the maintenance of the balance between tolerance and immunity, and the outcome of an immune response is determined by the type of released cytokines [24]. The ability of DCs to induce both pro- and anti-inflammatory responses is due to the existence of various recently discovered regulatory mechanisms, including the cross-talk between Wnt–β-catenin and Toll-like receptor (TLR) cascades [25]. A study indicates that both Wnt signaling and Notch signaling promotes differentiation into DCs in humans and mice, both in vivo and in vitro [26]. Notch signaling is an upstream regulator of the Wnt pathway in HSCs via the upregulation of the FZD family of Wnt receptors, which is regulated by the CSL (RBP-Jκ) transcription factor [26]. Co-receptors LRP5 and LRP6 are critical mediators of the canonical Wnt signaling pathway [2,27]. On the other hand, specific ablation of LRP5 and LRP6 in DCs is associated with delayed tumor progression and enhanced host anti-tumor immunity [28]. The Wnt pathway conditions DCs into a regulatory state when they surround a tumor microenvironment (TME) and suppress host immune activity against the tumor [29]. Some members of the Wnt signaling family Wnt3a, Wnt5b, and Wnt16 activate the β-catenin–TCF pathway in DCs [24]. The activation of Wnt3a or deletion of GSK-3β also stimulates the Wnt–β-catenin pathway and the specific inhibitor SB216763 suppresses GSK-3β activity [30].

A study has been conducted to further discern the effect of the Wnt–β-catenin signaling pathway on regulatory versus inflammatory responses, where specific ablation of β-catenin in DCs increased inflammatory responses. Consequently, β-catenin is associated with a tolerogenic state and helps to control colitis in a murine model [31]. Accordingly, the mechanism behind immunological tolerance driven by DCs attributed to β-catenin has been elucidated by expression of vitamin A–metabolizing enzymes and interleukin 10 (IL-10) in combination with such signaling molecules as Fas, phosphoinositide-specific phospholipase C (PLCγ2), mitogen-activated protein kinases (MAPKs), TLRs, and PI3K with AKT [32,33,34,35]. An alternate method of β-catenin–controlled immunological tolerance mediated by DCs is the induction of a specific disruption in the interactions involving homophilic *E*-cadherin [36]. Although the ablation of homophilic *E*-cadherin induces maturation of DCs, it inhibits the production of proinflammatory cytokines, thus resulting in the differentiation of progenitors into IL-10–producing cluster of differentiation 4–positive (CD4^+^) T cells. In a murine model, Wnt3a and Wnt5a induce activation of a tolerogenic response among DCs by altering their responses to lipopolysaccharide; on the contrary, they do not cause any changes in DC maturation [37]. Moreover, a melanoma-derived Wnt ligand (Wnt5a) facilitates the activation of β-catenin signaling, which causes an increment in the production of indoleamine 2,3-dioxygenase (IDO) in DCs, leading to the increased production of regulatory T cells [38,39].

In cancer, immunosuppression is promoted by dysfunctional DCs in the TME [40,41]. Increased levels of Wnts in the TME can trigger paracrine signaling and regulate host antitumor immunity [42,43]. In a TME, DCs metabolize vitamin A to retinoic acid. In addition, high levels of IL-10 and TGFβ expressed by DCs induce immune tolerance through CD4^+^ and CD8^+^ T cells [27]. Of note, IL-10 production is dependent on β-catenin/TCF4 and mTOR pathways [28,44]. Furthermore, a recent study suggests that Wnt1 induces a tolerogenic response in lung adenocarcinoma. Wnt1 inhibits CC and CXC motif chemokine transcription in DCs via the downregulation of transcription factor Cebpb, which is inversely related to T-cell abundance and thereby induces a tolerogenic response in lung cancer [45]. As discussed above, enhanced Wnt signaling promotes tumor progression and may contribute to immune evasion. Thus, further studies are needed to investigate distinct ways to alter Wnt signaling in tumor cells; this approach may cause beneficial changes in the TME and improve cancer treatment. 

#### 3.1.2. Wnt Signaling in NK cells 

The Wnt–β-catenin signaling pathway has been implicated in the development of NK cells and activates natural killer T (NKT) cell development and function [46]. Wnt3a primarily signals via the β-catenin dependent pathway, and a study indicates that exposure to Wnt3a or lithium chloride enhances NK-cell production and changes the differentiation potential of human thymic progenitors [47]. In contrast, the coculturing of human CD34^+^ CD38^−/low^ HSCs with OP9 stromal cells expressing Wnt3a induces a reduction in the number of NK cells isolated from these HSCs [48]. Nonetheless, active Wnt3a signaling in OP9 stromal cells causes an alteration in the transcriptional profile of these stromal cells, suggesting that the variation in the stromal-cell phenotype could contribute to the reduction in the differentiation into NK cells [48]. The altered outcomes of NK-cell differentiation because of Wnt3a expression highlight the role of other (unidentified) ligands and pathways that are yet to be explored. Human CD34^+^ HSCs when cultured under the conditions favorable for differentiation into NK cells with hydrocortisone, stromal cells, and cytokines (IL-3, IL-7, IL-15, Fms-related tyrosine kinase 3 ligand [FLT3L], and stem cell factors) show increased mRNA expression of LEF1 and TCF1 in contrast to suboptimal differentiation conditions with cytokines only [46,49]. Nonetheless, any blockage of the Wnt signaling pathway negatively influences their differentiation into NK cells. For example, the introduction of DKK1, which is a natural inhibitor of β-catenin dependent Wnt signaling, leads to a reduction in the number of NK cells, which normally develop under these conditions [46]. Another factor, such as DKK1, DKK2, or DKK4, inhibits the Wnt signaling pathway, where DKK3 does not show any affinity for LRP6, and for this reason, its function in Wnt signaling remains elusive [50]. Furthermore, a mouse study has revealed that both TCF1 and LEF1 contribute significantly to NK-cell development, and TCF1 plays a more prominent part in NK-cell development [51]. β-Catenin–deficient mice have a decreased number of NK cells in vivo as compared to the control—these data are suggestive of an important function of β-catenin, LEF1, and TCF1 in HSCs [52].

Moreover, CD1D-restricted NKT cells have a crucial role in tumor rejection and immune regulation and are dependent on the regulatory relation between LEF1 and the *CD1D* gene. A study on two leukemia cell lines-Jurkat cells (T lymphocytes) and K562 (myelogenous leukemia cell line) has unveiled the underlying mechanism of interaction in which LEF1 specifically binds to the *CD1D* promoter and regulates *CD1D* expression [53]. Nevertheless, an activated NKT cell produces cytokines that can regulate other immune cells (DCs, NK cells, and T cells) surrounding the TME by secreting IL-4 and IFNγ thereby implementing anti-tumor responses [54]. 

#### 3.1.3. Wnt Signaling in T cells

T cells are a type of lymphocyte that, according to recent findings, have a significant function in CD4^+^ and CD8^+^ T-cell–mediated adaptive immune responses. In the case of viral infection, naïve T cells trigger the formation of T effector cells that are detrimental to pathogens via cytotoxicity and also form memory T cells, which respond more efficiently to any future infection [55]. Memory T cells downregulate the activity of T effector cells in an antigen-independent manner by utilizing IL-7 and IL-15 [56]. On the contrary, in cancer, T cells become dysfunctional due to consistent exposure to an antigen in the TME and start to express inhibitory receptors, including LAG-3, Tim-3, CTLA-4, and PD-1 [57,58].

In T cell development and regulation, the contributing pathways include Wnt/β-catenin, SMAD, signal transducer and activator of transcription 3 (STAT3), and Notch signaling pathways [59]. Nevertheless, the first evidence of the participation of Wnt signaling in the immune system originates from the studies on T-cell development in the thymus [3]. Wnt signaling has been reported to perform a significant function in thymopoiesis. During the initial phases of thymocyte development in mice, high-mobility group (HMG) transcription factors of the Wnt pathway (TCF1 and LEF1) are known to be essential for the regulation of thymocyte development and maturation [60,61]. Precursor T cells mature in the thymus, owing to the presence of Delta-like ligands for Notch, which is essential for T-cell development in humans and mice [62,63]. One of the downstream target genes of Notch signaling is TCF1, which subsequently restrains LEF1 to stop the transformation of thymocytes; in TCF1-deficient mice though, it stimulates T-lineage maturation [64]. By direct ablation of double-positive (DP) thymocytes, researchers have found that TCF1 and LEF1 deficiency diminishes the maturation of CD4^+^ T cells into the CD8^+^ cell lineage. Both TCF1 and LEF1 interact with β-catenin to regulate the DP (CD4^+^CD8^+^) cell differentiation into CD4^+^ T cells, and in this process, Th-POK is an upstream regulator. In contrast, CD8^+^ T-cell maturation and development are regulated by the crosstalk between TCF1 and RUNX3 serving to silence *CD4* gene expression [65]. Histone deacetylases HDAC1 and HDAC2 and transcription factor Th-POK are reported to maintain the integrity of CD4^+^ T cells by repressing the genes associated with the CD8^+^ lineage [66,67]. Similarly, a study has revealed that Wnt transcription factor LEF1 and TCF1 are important for establishing CD8^+^ T-cell identity due to HDAC activity, by downregulating RAR-related orphan receptor C (RORC), forkhead box P3 (FOXP3), and CD4 in a mouse model [68]. Moreover, TCF1 has multiple isoforms in which it possesses a long chain of the β-catenin N-terminal domain. The crosstalk between β-catenin and the long N-terminal domain maintains thymocyte survival instead of thymic maturation as identified in TCF1 isoform–deficient (p45^−/−^) mice [69]. During thymopoiesis, the importance of β-catenin has been confirmed because it upregulates interleukin 7 receptor subunit α (IL7R-α) in thymocytes through positive selection [70]. A study has shown that by means of soluble FZD-type receptors as a decoy, thymocyte development can be stopped in murine thymic organ culture possibly owing to the disruption of Wnt signaling [71]. Both TCF1 and LEF1 are necessary because targeted gene disruption completely blocks thymocyte differentiation. Similarly, in another study, double mutation LEF1^-/-^TCF1^-/-^ in mice induced T-cell differentiation arrest at an immature CD8^+^ single-positive stage, in cells expressing T-cell receptor beta (TCRβ) but with reduced TCRα gene transcription [72]. The transition of thymocytes from double-negative (DN) to DP is regulated by Wnt signaling. The expression of naturally occurring inhibitor of β-catenin and TCF (ICAT) blocks the thymocyte transition from the DN to DP stage, but does not have any effect on later developmental stages. On the contrary, DKK1 inhibits the binding of Wnt to co-receptors LRPs and stops the thymocyte differentiation in a dose-dependent manner at the DN stage [73,74]. As for β-catenin, its function in CD8^+^ T cells is to induce stem cell–like properties (self-renewal and differentiation into effector cells); similarly, TCF1 induces the same functionality in these cells, and silencing of its expression eliminates the stem cell–like properties from CD8^+^ T cells [75]. Accordingly, the Wntβ-catenin pathway positively correlates with the progression of a tumor and metastasis. It is a significant oncogenic pathway that induces immune evasion and is thereby negatively associated with the effector CD8^+^ T cell infiltration at the tumor site [59].

In a mouse model of lymphocytic choriomeningitis mammarenavirus chronic infection, among CD4^+^ T cells, memory cell (Th1, Tfh) production is regulated due to the presence of TCF1 long isoforms [76]. Moreover, the induction of the GATA-3-1b isoform by TCF1 affects the differentiation of Th2 cells, and its absence can protect mice from ovalbumin-induced asthma [77]. Wnt3a has been found to activate Th2-cell differentiation via β-catenin and special AT-rich sequence binding protein 1 (SATB1) [78]. In contrast, Th17 cells have been demonstrated to eliminate tumors and to express large amounts of TCF7 and β-catenin while manifesting the characteristics similar to those of early memory CD8^+^ T cells [79]. Microarray analysis of chemokine ligand CXCL has confirmed that the expression of Wnt5a in human CD4^+^ T cells is necessary for T-cell migration [80]. 

#### 3.1.4. Wnt Signaling in B cells

The Wnt pathway is associated with the regulation of various essential cellular processes including lymphopoiesis, although its role in B cells is perhaps less understood, especially the role in B-cell progenitors in bone marrow as compared to Tcells in the thymus [3]. The early stage of B-cell growth is regulated by canonical and non-canonical Wnt pathways; however, its aberrant activation has oncogenic complications [81,82]. B-cell proliferation is regulated by Wnt signals through LEF1, and a study revealed that mice deficient in LEF1 have defects in pro-B-cell proliferation and survival both in vivo and in vitro. Due to increased *c-Myc* and *Fas* transcription, the sensitivity to apoptosis is higher [83]. FZD9^−/−^ mice have a defect in B-cell lymphopoiesis; this defect negatively affects B-cell development in bone marrow, especially in cycling pre-B cells [84]. Nonetheless, in the case of human B-cell progenitors, Wnt3a stimulation negatively affects the proliferation potential of B cells despite increased β-catenin levels [85]. Similarly, there is antagonism between canonical Wnt signaling and Wnt5a signals in the thymus because Wnt5a signals via noncanonical pathways, thereby inhibiting the proliferation of B cells in a cell-autonomous manner. On the other hand, the absence of the wild-type Wnt5a allele induces B-cell lymphomas and clonal myeloid leukemia in hemizygous mice. Wnt5a gene deletion or loss of its expression has been observed during the analysis of human primary leukemia [86]. Similarly, Hodgkin lymphoma (HL) originates from transformed Reed–Sternberg (RS) cells, which usually lack B-cell receptor expression [87]. Additionally, HL cell lines express other components of the Wnt pathway and increased levels of cytoplasmic and nuclear β-catenin [88,89]. Nevertheless, the activation of GSK-3β in classical HL (cHL) consequently inhibits Wnt-β-catenin signaling [90].

In a TME, aside from the production of cytokines and antibodies, B cells play a versatile role in the modulation of innate immune and T-cell responses [91]. By contrast, tumor-infiltrating B lymphocytes have been identified in solid tumors and play a significant role in cancer suppression by releasing immunoglobulins and activating T cells to directly kill cancer cells [92]. The expression of specific markers on different B-cell subtypes has contradictory effects on pro- or antitumorigenic processes. A recent study revealed that during an early phase of tumor progression, B cells produce antibodies that cause DCs and cytotoxic T cells to control tumor growth [93]. On the contrary, regulatory B cells promote a pro-tumorigenic response to facilitate tumor progression. Notably, a study has been performed on acute myeloid leukemia (AML) where B cells include a higher proportion of regulatory B cells and display surface markers CD19, CD24, and CD38 [94]. Nonetheless, less attention is given to the investigation of the role of Wnt signaling in B-cell development and regulation along with its participation in carcinogenesis. Therefore, further research is needed in the context of B cells to develop new treatment modalities for cancer.

### 3.2. Wnt Signaling in Myeloid-Originated Immune Cells

#### 3.2.1. Wnt Signaling in Macrophages

Macrophages are essential for homeostasis in most of organ systems for tissue repair and development; additionally, these cells provide defense against pathogens, cancer, and chronic inflammation [95]. The involvement of macrophages in a diverse array of cellular functions gives these cells plasticity to adapt well to their microenvironment [96]. In many organs after tissue injury, macrophages establish a tissue repair program, and the associated Wnt ligands are essential players in tissue regeneration and fibrosis [97]. Wnt-specific ligands also play a regulatory part during infection or inflammation. It is speculated that in response to isolated TLR ligands, NF-κB activation, or *Mycobacterium tuberculosis*, macrophages express Wnt5a and its receptor FZD5 [98]. Similarly, the expression of Wnt5a and FZD5 has been observed in biopsy samples from patients with sepsis [99]. Another study revealed bacterial infection (with *Francisella tularensis*) in murine peritoneal macrophages that were caused by the activation of GSK3β both in vivo and in vitro. Inhibition of GSK3β leads to a noticeable reduction in the production of anti-inflammatory cytokines such as tumor necrosis factor α (TNFα), IL-12p40, and IL-6 [100]. According to these results, GSK3β acts as a regulator that modulates the inflammatory responses and could be harmful to the host during infection by *F. tularensis*, thereby pointing to novel therapeutic targets in tularemia. Macrophages and Wnt proteins are involved in cardiac repair as observed in mice after myocardial infarction [101]. A study on mice has revealed that the specific ablation of the Wntless (WLS) protein, which is essential for the secretion of Wnt ligands, improves heart function and cardiac repair after an ischemic injury caused by myocardial infarction [101]. These findings point to a potential therapeutic target for the improvement of cardiac repair via targeting of natural Wnt inhibitors. The intestinal stroma is mainly composed of macrophages, which have been found to perform a pivotal function in the coordination of intestinal repair because of the presence of macrophage-derived Wnt signaling. To further unravel the macrophage-derived Wnt signaling, porcupine *O*-acyltransferase (PORCN) depleted mice have been studied and show normal intestinal morphology but are hypersensitive to radiation injury as compared with wild-type littermates [102]. In acute murine colitis treated with 2,4,6-trinitrobenzene sulfonic acid, the STAT6-dependent macrophage phenotype mediates mucosal repair via activation of the Wnt signaling pathway [103]. In a STAT6^−/−^ murine model treated with 2,4,6-trinitrobenzene sulfonic acid, impaired wound healing was observed and yielded a reduction in mRNA expression of Wnt ligands (Wnt2b, Wnt7b, and Wnt10a) in cells of the lamina propria and mucosa [103]. Furthermore, Wnt7b has a repair function because its somatic deletion in macrophages negatively influences tissue repair and kidney regeneration after ischemic injury in a mouse model [104]. The expression of the Wnt7b protein in myeloid cells induces tumor progression, metastasis, angiogenesis, and enhances the functionality of tumor-associated macrophages (TAMs) in humans and mice. In myeloid cells in an MMTV-PyMT mouse model, *Wnt7b* gene deletion induces a dramatic reduction in mammary gland tumor volume and mass as compared to wild-type mice [105]. On the other hand, it has been reported that the induced expression of Wnt3a causes macrophages to engulf hepatocyte debris during liver regeneration [106]. This process activates the commitment of murine hyperspiny purkinje cells to the hepatocytes through the β-catenin–dependent pathway in a mouse model [106]. Wnt5a is important for macrophage-induced invasiveness because of its proteolytic activity, along with its ability to regulate tumor cell migration, insulin resistance, atherosclerosis, and obesity [107,108].

A proinflammatory activity of Wnt5a in macrophages has been demonstrated too, after the identification and internalization of Chandipura virus or *Escherichia coli* by receptor CD14. The Wnt5a–FZD5–Rac1–p65 signaling cascade consequently activates TLR signaling in bone marrow-derived macrophages and in a murine macrophage (RAW 264.7) cell line [109]. Wnt5a induces a proinflammatory response not only during infection and organ repair or injury; for example, a study suggests that Wnt5a may activate an immunosuppressive response in macrophages in both humans and mice [110]. In humans, the differentiation into M1-type macrophages is inhibited via Wnt5a-induced suppression of the NF-κB pathway. Therefore, this triggers the production of immunosuppressive cytokines (e.g., TGF-β and IL-10) and induces an M2 macrophage like phenotype [110]. The M2-like phenotype acquired by TAMs conducive to the production of inflammatory cytokines IL-23 and IL-17, which have been reported to positively promote tumor growth and progression [111]. Tumor cells have been demonstrated to reprogram macrophages into a distinct TAM population via the exosomal pathway. This pathway plays an important role in the transport of miR-1246 from P53-mutant colon cancer cells to peripheral macrophages—this process induces M0 and M2 macrophages to produce TGF-β, IL-10, VEGF, and CCL2 [112]. 

In brief, the actions of Wnt ligands in macrophages can significantly participate in the repair of tissue injury, although the reparative function is not universal. This is because not all Wnt ligands have the same biological activities and functions. Therefore, to obtain full mechanistic insight into each Wnt ligand’s activity and its signaling components, additional studies are necessary, which will further clarify the participation of Wnt in various biological functions [110].

#### 3.2.2. Wnt Signaling in Granulocytes

Granulocytes (mast cells, neutrophils, eosinophils, and basophils) contribute significantly to inflammation both in immune regulation and pathogen removal; they differentiate and mature in bone marrow before entering the blood circulation where they remain in the G0 phase in the absence of extracellular stimuli and undergo apoptosis [113,114]. As for Wnt signaling, it has been identified as a key process in the function of granulocytes. However, the mechanism is less studied. Moreover, neutrophils are among the first lines of defense because they are recruited to an area of infection or injury, and Wnt5a mediates this process [115]. By contrast, in eosinophils, Wnt5a in combination with other factors promotes cell proliferation, as observed in airway smooth muscle cells isolated from asthma patients [116]. Here, we briefly focus on mast cells.

Mast cells are the type of immune cells that reside in connective tissue throughout the body and significantly partake in the regulation of adaptive and innate immunity [117]. These cells originate from HSCs and differentiate within the tissue environment upon migration to peripheral tissues [118]. Nevertheless, an interesting similarity between mast cells and HSCs has been uncovered, including c-Kit expression and self-renewal. Wnt5a has been identified as a significant factor in this context because Wnt5a promotes the maturation of mast cells through the Wnt-β-catenin pathway [119]. Furthermore, mast cells contribute as a promoter and inhibitor of tumor growth, but the exact mechanism is not well understood [120]. The production of proangiogenic factors such as IL-8, TNF-α, vascular endothelial growth factor (VEGF), basic fibroblast growth factor (bFGF), and transforming growth factor β (TGF-β) promotes angiogenesis, whereas anticancer mediators include IL-1, IL-6, chymase, TNF-α, and tryptase [120]. Further studies are needed to explore the activities of Wnt in granulocytes for the development of therapeutics.

## 4. Wnt Signaling in Cancer

**Figure 3:**
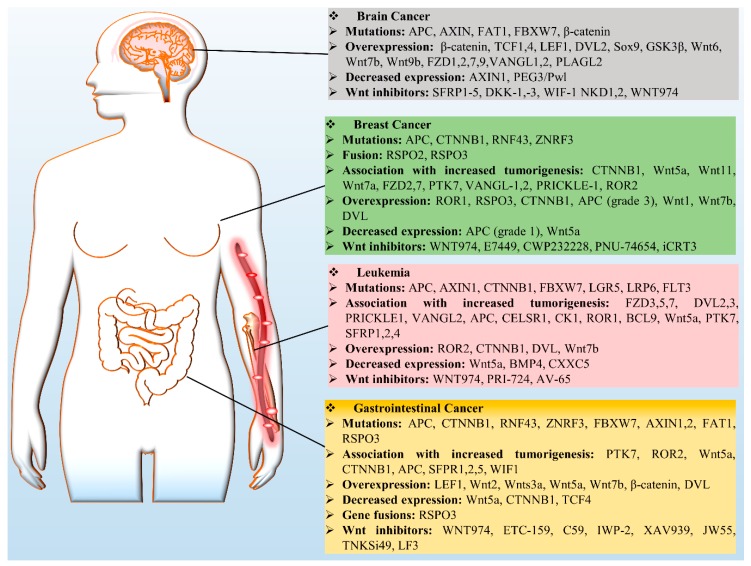
Wnt signaling in cancers. Deregulation of Wnt signaling components is involved in selected cancers (brain cancer, leukemia, breast cancer, and GCs) via a variety of mechanisms, including mutations, overexpression of Wnt proteins, decreased expression, gene fusion, and increased tumorigenesis; these data are summarized in the boxes. Protein symbols: BCL9, B-cell CLL or lymphoma 9; BMP4, bone morphogenetic protein 4; CELSR1, cadherin EGF LAG seven-pass G-type receptor 1; FBXW7, F-box and WD Repeat Domain -containing 7; FLT3, Fms-related tyrosine kinase 3; NKD1, naked cuticle homolog 1; PEG3/Pwl, paternally expressed 3; PLAG2, pleiomorphic adenoma-like protein 2; SFRP1, secreted frizzled-related protein 1; VANGL, Vang-like protein; WIF-1, Wnt-inhibitory factor 1.

Wnt signaling has been identified as a key player that governs several developmental and cellular processes such as cell proliferation, migration, and fate determination. Nonetheless, slight variation in this pathway could be detrimental by inducing cancer and therefore has been recognized as a key mechanism in cancer biology. There are several aberrant regulatory processes affecting Wnt signaling components, e.g., mutations, overexpression, and downregulation (Figure 3). There are numerous studies on the role of Wnt in cancer, however in this review we mainly focus on leukemia and breast, brain, and GCs.

### 4.1. Breast Cancer

Wnt signaling is activated in approximately 50% of breast tumors and is associated with reduced overall survival of the patients [121]. Wnt–β-catenin signaling is deeply involved in the initiation and progression of triple-negative breast cancer (TNBC) [122,123]. However, other breast cancer subtypes overexpress nuclear β-catenin [124]. Wnt signaling not only induces breast development during pregnancy, but also plays a crucial part in the oncogenic transformation of mammary tissue. The overexpression of FZD7 and Wnt7a is triggered by ∆Np63, which then activates stem cells that promote basal-like breast cancer [125]. The overexpression of R-spondin 2 initiates mammary tumors in mouse models [126]. Furthermore, ROR1 is not present in normal mammary tissue, but is overexpressed in breast cancer cells. Thus, it leads to rapid epithelial–mesenchymal transition and is linked to a low survival rate [127]. The up-regulation of PD-L1 in TNBC cancer stem cells is therefore associated with *Wnt* gene activation. Furthermore, clinical dataset analysis has revealed PD-L1 overexpression in TNBC tumors: a tradeoff between more enriched stem cells and enhanced Wnt activity [128,129].

Besides, further updates have uncovered a relationship between resistance to a PI3K inhibitor and the upregulation of the canonical Wnt pathway—these phenomena are associated with poor prognosis among breast cancer patients [130]. Additionally, Wnt1-inducible signaling pathway protein 1 (WISP1) can transcriptionally block N-Myc downstream-regulated gene 1 (NDRG1) resulting in breast cancer metastasis [131]. Wnt pathway-driven processes can cause population heterogeneity of mammary breast cancer cells, among which the luminal subtype secretes Wnt1, which is essential for the propagation of a tumor and for cancer recurrence [132]. Nevertheless, deficiency of LRP5 results in a delay in Wnt1-triggered tumorigenesis followed by a reduction in progenitor cell accumulation [133]. On the other hand, DKK1 or DKK3 deficiency induces the self-renewal of progenitor cells by initiating the β-catenin pathway [134]. In human breast cancer, the CD44^(+)^ CD24^(−/low)^ lineage shows high tumorigenicity, and both canonical and noncanonical Wnt signaling plays a crucial role in the implementation of epithelial–mesenchymal transition (EMT) and in the stem cell phenotype [135]. 

A Wnt pathway crosstalk is substantial in breast cancer; for example, in the Hippo pathway, a transcriptional coactivator called WW domain binding protein 2 (WBP2) forms a network with YAP, TAZ, and β-catenin, which then promotes TCF-induced malignancy [136]. These findings suggest that the presence of a polyclonal cell population in mammary tumors is due to Wnt activity in a specific subpopulation.

### 4.2. Leukemia 

In recent years, it has become clear from various findings that the disruption of the Wnt pathway is important for the progression of hematological cancers [137]. Normal HSCs rely on the controlled regulation of Wnt signaling because it is a critical regulator of differentiation and self-renewal, whereas increased Wnt pathway activity is found in major leukemia [22]. AML is one of the most common leukemia with balanced translocations t(8;21) [138]. In AML, galectin-9-induced autocrine T-cell immunoglobulin mucin-3 (TIM-3) signaling stimulates LRP6 signalosome formation and the accumulation of β-catenin in the nucleus [139]. Accordingly, exosomes extracted from the plasma of AML patients show high levels of TGF-β1 and suppress NK-associated cytotoxicity [140]. Notably, tumor-derived exosomes are critically involved in the suppression of immune cells by utilizing CD8+ anti-tumor effector cells. An exosomal profile can be used for disease prognosis and facilitates the characterization of a therapeutic outcome.

In mixed-lineage leukemia fusion positive mouse model, HSCs give rise to myeloid progenitor cells and leukemia-initiating cells (LICs) after progression via pre-LIC state [141,142]. β-Catenin is crucial for the progression from a pre-LIC to the LIC state and for the self-renewal of LICs [141,143]. Chromosomal aberrations affect canonical Wnt signaling because of frequent AML translocations (PML-RARα, AML1-ETO, and mixed-lineage leukemia [MLL]-AF9), which have been identified in derived cell lines and clinical samples [142,144,145]. The crosstalk of Wnt and NOTCH also promotes leukemic transformation of HSCs because of an interaction of β-catenin with forkhead box protein O1 (FoxO1) in osteoblasts which upregulates jagged1 [146]. 

The most common type of childhood leukemia is acute lymphoblastic leukemia (ALL), where bone marrow is infiltrated by immature lymphoblasts expressing T-cell immunophenotypic surface markers [147]. As for T cells, in ALL, most LICs harbor mutations that activate the Notch signaling pathway. Furthermore, the presence of canonical Wnt signaling in thymocytes and HSCs along with *c-Myc* amplification induces β-catenin-dependent and Notch-independent activation of T-cell acute lymphoblastic leukemia (T-ALL) [148,149]. In Notch1-induced T-cell leukemia, the presence of high proportions of leukemic stem cells correlates with activated Wnt signaling [150]. Canonical Wnt signaling not only specifically induces tumorigenesis in T-ALL subsets but also takes part in LIC self-renewal. A study on a mouse model of Notch1-mediated T-ALL confirms that LICs feature increased Wnt activity and genetic inactivation of β-catenin causes a reduction in the number of LICs in these tumors [150]. 

Chronic lymphocytic leukemia (CLL) is the most common hematological cancer and is characterized by the accumulation of dysfunctional but morphologically mature CD5+ cells [151]. In CLL cells, canonical Wnt signaling is active, whereas its inhibition induces apoptosis in vitro [152]. Apart from the silencing by a Wnt-inhibiting factor, such as DKK1 or DKK2 [153], the mutation in a Wnt pathway-associated gene (for example *BCL9* or *FZD5*) accounts for 14% of studied cases [154]. The overexpression of Wnt3, Wnt5b, Wnt6, Wnt10a, Wnt14, Wnt16, LRP5 and LRP6, LEF1, ROR1, or receptor FZD3 in CLL has been reported, contrary to normal B cells [152,155,156]. These findings validate our notion that the survival of CLL cells relies on active Wnt signaling. Moreover, the exosomes surrounding a CLL cell microenvironment contain noncoding Y RNA hY4 in abundance. These exosomes can be utilized by monocytes to activate the TLR cascade and secrete large amounts of CCL2, CCL4, IL-6, and PD-L1 to promote CLL progression [157].

Multiple myeloma has been characterized as the second most common hematological cancer associated with the aberrant Wnt signaling. Nonetheless, no oncogenic Wnt pathway mutation has been identified, indicating Wnt signaling stimulation in an autocrine or paracrine manner in a bone marrow microenvironment [158]. In hematological cancers, the cross-talk between innate and adaptive immunity is critical [159], and a deep understanding of this relation will facilitate the development of new therapeutics. In major leukemia, the presence of active canonical Wnt signaling is important for tumor initiation, progression, maintenance, and for the survival of LICs.

### 4.3. Gastrointestinal Cancers (GCs)

These cancers are among the most prevalent malignant tumors worldwide and are a prominent cause of cancer-related deaths [160]. The causative mutations found in the majority of colorectal tumors are associated with the following pathways: Wnt, TP53, PI3K, MAPK, and TGF-β [161,162]. Accordingly, other mutations in the Wnt pathway are also associated with CRC, including transcription factor 7–like 2 (TCF7L2), Wilms tumor gene (FAM123B), and CTNNB1 [163,164]. The mutation driving Wnt signaling in CRC is the loss of the *APC* gene [165]. The oncogenesis of CRC can be modeled ex vivo in human-engineered intestinal organoids by means of the genome-editing CRISPR/Cas9 technology [166,167]. Moreover, studies on CRC tumors have uncovered different APC mutations at specific levels of the canonical Wnt pathway, and these mutations are associated with particular tumor locations within the proximity of the large intestine [168,169]. CRC cells can regain their normal function upon a reversible knockdown of APC with short hairpin RNA [170]. In contrast, exosome-mediated communication in the TME also promotes tumor progression. Patients with colon cancer show a higher concentration of miR-203 in plasma exosomes, which triggers the differentiation of monocytes into M2-TAMs, consequently providing a favorable environment for tumor progression [171]. Accordingly, TAMs are regulated by tumor-derived exosomes, which influence host immune function and promote tumor spread to distant organs.

Hepatocellular carcinomas primarily harbor a missense mutation or insertions/deletions within *CTNNB1* exon 3, thereby inducing defects in the production of β-catenin, which remains hypophosphorylated at Thr41, Thr45, Ser33, and Ser37 and hence is undegradable [165]. *Helicobacter pylori* infection is another major causative factor of GCs because it promotes Wnt–β-catenin signaling by activating cytotoxin-associated gene A (CagA), which induces cancer stem cell–like characteristics in GCs as β-catenin specifically binds to the promoter regions of OCT4 and NANOG [172]. Additionally, H. pylori–induced human GC is also associated with SOX9 expression, which eventually makes cancer cells responsive to β-catenin–dependent signaling [173]. Erythropoietin-producing hepatoma receptor A2 (EphA2) has been reported to enhance β-catenin nuclear localization and induces *c-Myc* transcription, whereas Dvl2 mediates EphA2–axin1 signaling by interacting with the tyrosine domain of EphA2 in GC cells [174]. Noncanonical signaling through Wnt5a also substantially partakes in GC progression: leucine-rich DVL associated protein mediates Wnt5a-triggered laminin γ2 expression via JNK and Rac1 stimulation [175].

The activation of Wnt signaling has been detected in cholangiocarcinoma, and the validated mutations identified so far are an inhibitory ring finger protein 43 (RNF43) mutation, overexpression of Wntless, and hypermethylation of secreted frizzled-related protein 2 (*SFRP2*) [176,177,178]. Furthermore, osteopontin, a chemokine-like glycoprotein, has been found to enhance Wnt signaling in cholangiocarcinoma upon activation of the MEK–MAPK1 pathway via Ser675 phosphorylation and nuclear localization of β-catenin [179]. Wnt-secreting macrophages are present around the TME to maintain strong Wnt signaling [180,181]. The activity of SFRP2, a Wnt signaling inhibitor, is silenced in cholangiocarcinoma because of the hypermethylation of its gene [178,182]. Recently, immunohistochemical analysis was performed to evaluate the expression of FZD5, CK1, Wnt5a, axin, GSK3β, ubiquitin, *c-Myc*, and cyclin D1—the results obtained are still unclear. Nonetheless, higher expression of components of the canonical Wnt pathway has been identified in samples of human gastric carcinomas [183]. 

### 4.4. Brain Cancer

The Wnt–β-catenin signaling pathway contributes significantly to various stages of brain development and remains crucial in the adult brain [184]. In neuro-oncology research, the mechanism of the Wnt pathway at a molecular level has piqued much interest among investigators. Wnt signaling controls and regulates anterior–posterior axis formation and neural differentiation during early vertebrate development [185]. By contrast, abnormal Wnt signaling in neural stem cells (NSCs) stimulates malignant transformation and initiates the formation of brain tumors [186]. For example, the prolonged activation of β-catenin augments neural progenitor cell proliferation in vivo, whereas its deletion reduces their proliferative capacity [187,188]. Additionally, Wnt3a upregulates Wnt signaling, and thus the clonogenic potential of NSCs increases [186]. Glioblastoma multiforme (GBM) is among the most lethal and common central-nervous-system tumors. GBM is resistant to treatment because it is distinguished by enhanced cell proliferation and extensive angiogenesis. The prevalent mutation characterized in GBM is homozygous deletion of FAT atypical cadherin 1 (*FAT1*), which initiates the upregulation of Wnt signaling in glioma. Similarly, another known feature of the GBM pathogenesis is the overexpression of the hepatocyte growth factor (HGF) pathway [189,190,191,192]. The data obtained within The Cancer Genome Atlas Program revealed that FAT1-inactivating mutations account for 1% of GBM cases, whereas approximately 20% of the cases are reported to be related to a copy number loss of FAT1 in GBMs. In addition to genetic aberrations, epigenetic alterations have been observed too, for instance, the hypermethylation of *SFRP1*, *SFRP2*, and naked 2 (*NKD2*) has been characterized and is present in more than 40% of primary-GBM specimens [193]. Moreover, abnormality of the *SFRP* gene expression is associated with the downregulation of matrix metalloproteinase 2 (MMP-2) and affects GBM cell motility [194,195]. The overexpression of pleomorphic adenoma gene–like 2 (PLAGL2) suppresses the activity of glioma-initiating cells and NSCs while stimulating their self-renewal potential. Furthermore, transcriptomic data suggest that the differentiation-suppressive effect is associated with PLAGL2 owing to variation in Wnt pathway components (Wnt6, FZD2, and FZD9) [196]. 

It is well known that Wnt signaling makes a significant contribution to the progression and regulation of characteristics of brain-associated cancers, and further insight into the molecular mechanism will help to explore the oncogenic effects of this pathway.

## 5. Therapeutic Targets in the Wnt Signaling Pathway

In the past, several studies have shown that the aberrant regulation of the Wnt signaling pathway is responsible for the initiation and progression of various immune disorders and cancers, e.g., osteoarthritis, asthma, and CRC in addition to prostate, lung, breast, and thyroid cancer, and CLL [2,159,197,198]. Accordingly, several studies are underway to identify therapeutic targets in the Wnt signaling pathway and to devise new therapies that either antagonize intracellular signaling or block extracellular signals by means of kinase inhibitors, (e.g., small-molecule inhibitors, antibodies, antagonists, or peptides; Table 1). With respect to therapeutic targets, Wnt signaling antagonists are classified into four major classes: ligand/receptor inhibitors, PORCN inhibitors, tankyrase (TNKS) inhibitors, and β-catenin inhibitors.

As for Wnt signaling, ligand/receptor-targeted drugs binding to ligands or transmembrane proteins represent the main therapeutic approaches that are evaluated in clinical studies on various diseases, cancer in particularl. A large number of drugs are reported, and most of them are monoclonal antibodies (moAbs) such as vantictumab (anti-FZD1, anti-FZD2, anti-FZD5, anti-FZD7, and anti-FZD8) [199], IgG-2919 (anti-FZD5) [200], OTSA101 (an anti-FZD10 antibody–drug conjugate [ADC]) [201], MC-Val-Cit-PAB-MMAE (anti-LGR5 ADC) [202], cirmtuzumab (anti-ROR1) [203], PF-06647020 (anti-PTK7 ADC) [204], OMP-131R10 (anti-RSPO3) [205], sclerostin [206], OMP-54F28 (anti-FZD8) [207], and ROR1 chimeric antigen receptor (CAR) T cells. All these therapeutics are classified as Wnt ligand/receptor-targeted drugs [208]. PORCN is a key regulator of the Wnt signaling pathway and takes part in the palmitoylation and secretion of Wnt ligands in the endoplasmic reticulum. PORCN inhibitors show promise for the suppression of Wnt signaling and for the treatment of various cancers by blocking the secretion and oligomerization of Wnt receptors [209,210]. Several small-molecule PORCN inhibitors are being tested in clinical trials including LGK974 (WNT974) [211], inhibitor of Wnt production-2 (IWP-2) [212], WNT-C59 (C59) [213], RXC004 [214], and ETC-159 (ETC-1922159; Table 1) [215].

Moreover, the inhibitor IWP-2 is proposed for the treatment of cancer, especially CRC due to RNF43 mutations, whereas PORCN inhibitors WNT974 and ETC-159 are applicable to cancer stem cells [200,211,216]. Recent studies show that PORCN inhibitor RXC004 strongly increases the effectiveness of immuno-oncology agents such as anti-PD-1 checkpoint inhibitors and enhances immune responses [214]. Nevertheless, several important features need to be considered for future therapeutics, for example, the global blockage of all Wnt secretion, which will undermine gut homeostasis and the effects of canonical and noncanonical Wnt signaling [217,218].

The TNKS enzyme is a member of the poly (ADP ribose) polymerase (PARP) superfamily that adds ADP-ribose onto target proteins. TNKS inhibitors suppress PARylation and degradation of oncogenic signaling proteins such as axin, angiomotin (AMOT), phosphatase and tensin homolog (PTEN), and telomeric repeat-binding factor 1 (TRF1). As a result, the abundance of axin increases, and the overactivated destruction complex inhibits the WNT-β-catenin signaling pathway and telomere shortening and suppresses Yes-associated protein 1 (YAP)-dependent transcription, while PI3K signaling is repressed [257,258,259]. Wnt–β-catenin signaling is blocked by various TNKS inhibitors such as tankyrase inhibitor 49 (TNKSi49) [236], XAV939 [226], JW74 [260], AZ1366 [261], IWR-1 [234,262], NVP-TNKS656 [232], WIKI4 [237], G007-LK [233], and JW55 [231] in different tumor models. Additionally, TNKS inhibitors are effective in a combination therapy with inhibitors (API2) of AKT (also known as protein kinase B) or with a PI3K inhibitor (BKM120), MEK inhibitor (AZD6244), or an EGFR inhibitor (erlotinib or gefitinib); this combination therapy shows promise against the tumors where β-catenin is overexpressed [230,232,263,264]. Nevertheless, TNKS inhibitors have numerous substrates that are involved in important cellular processes like the regulation of telomere length, Wnt signaling, myelination, and lung fibrogenesis. Although the clinical effectiveness of TNKS inhibitors is high, this inhibition may have acute adverse effects, and these inhibitors have been delayed at the preclinical stage [265].

The interaction of β-catenin with transcription factors (TCF or LEF) is an irresistible therapeutic target; in particular, inhibition of β-catenin enhances its degradation and disrupts its binding to TCF or LEF [266]. There are many reported small-molecule compounds that antagonize protein–protein interactions such as, e.g., β-catenin–TCF, stabilized α helix of B-cell lymphoma 9 (SAH-BCL9) [244], BC-2059 [241], PRI-724 [267], CGP049090 [268], PKF115-584 [269], LF3 [242], CWP232228 [270], and MSAB [243]. Other than the above-mentioned compounds, a number of synthetic molecules such as inhibitors of catenin-responsive transcription (iCRT3, iCRT5, and iCRT14), PNU-74654, and BC21 have been identified by high-throughput screening and computational-docking studies [271,272,273]. Although these in silico and in vitro screening procedures produce a large number of molecules, the biological activity and binding mechanism for most of them are not clear [274]. Furthermore, there are numerous difficulties, for instance, the binding affinity of β-catenin for TCF is very strong (20 nM), and the role of β-catenin in cell adhesion is unknown where there is an overlap of *E*-cadherin and TCF interaction sites [225]. Therefore, novel strategies are needed to overcome these hurdles and to target the interaction of β-catenin with various transcription cofactors.

There are several recently discovered therapeutic targets such as dickkopf family members (DKK1, DKK2, DKK3, and DKK4), which perform a significant function as immunomodulators in immune diseases (as discussed earlier) [275]. The DKK1 receptor and cytoskeleton-associated protein 4 (CKAP4) are expressed in tumor cells, suggesting that the inhibition of DKK family members and DKK1–CKAP4 interactions can be examined as a potential therapeutic target, but further studies are still needed to validate this approach [276].

## 6. Conclusions and Future Directions of Research

The Wnt signaling pathway plays an essential role in cell differentiation, division, and proliferation, as well as embryonic growth, stem cell development, tissue regeneration, and immune cell regulatory mechanisms [1,2]. In this review, we broadly discussed the participation of Wnt signaling in immune cell modulation and the aberrant regulation of Wnt pathway components in cancer and highlighted the relevant therapeutic targets. There are various mutations as well as the overexpression and downregulation of Wnt pathway components: These aberrations can be regarded as causes of immunological disorders and cancers. The role of Wnt signaling in immune cells is becoming an area of active research because of the significant participation of this pathway in the regulation of immune cells apart from cellular survival, proliferation, and development. The functions of Wnt signaling in immune cell–regulatory mechanisms are diverse, for example, the tolerogenic response among DCs, development of NK cells, thymopoiesis of T cells, and B-cell–driven initiation of T-cell activities and Wnt signaling in macrophages. These phenomena are involved in tissue repair, regeneration, and fibrosis [3,26,46,97]. Moreover, all these processes are intricate and lack mechanistic illustrative studies—this situation precludes the invention and formulation of therapeutic strategies and convolutes the outcomes of existing therapeutic modalities. By examining the underlying mechanisms, we revealed that targeting of Wnt signaling can probably overcome all the primary, adaptive, and acquired resistance to cancer immunotherapy.

Therefore, drug monotherapy is usually insufficient for defeating cancer owing to the complex signaling mechanisms and interactions of pathways. We would like to emphasize that combination therapy is expected to be highly useful for targeting Wnt signaling during cancer treatment in order to help clinicians to attain a successful result. Although there are several Wnt inhibitors that have been studied regarding cancer treatment, it would be worthwhile to extend this research to immune cells.

## Figures and Tables

**Table 1:** Therapeutic targets and inhibitors that are evaluated in clinical studies regarding cancers and Wnt signaling.

Target	Compound Name	Cancer Model	Description	Activity (IC50)	Clinical Phase	Reference/Clinicaltrials.gov
	Small-molecule compounds					
PORCN	WNT974 (LGK974)	BRAF mutant colorectal cancer	In combination with cetuximab and LGX818	0.4 nM	Ib/II	NCT02278133
		Primary ovarian cancer (OV-7 and OV-14 cell lines)	In combination with carboplatin	1.14 µM, 1.76 µM	NA	[219]
		Head and neck squamous carcinoma	Reduced axin 2 mRNA level	0.3 nM	II	NCT02649530
		Triple-negative breast cancer	In combination with buparlisib. Dual targeting of the PI3K and Wnt pathways	NA	I	NCT01351103 [220,221,222]
		Melanoma	Antitumor activity	NA		
		Pancreatic adenocarcinoma	Reduces the expression of Axin2	NA		
	ETC-159 (ETC-1922159)	Solid tumors	Induces tumor regression	NA	I	NCT02521844
		Colorectal cancer with R-Spondin translocations	Prevents tumor regrowth by inducing irreversible cellular differentiation	2.9 nM	Preclinical	[215]
	C59 (WNT C59)	Mammary tumors in mice	Inhibits Wnt-1–promoted tumor growth in mice	74 pM	Preclinical	[213]
		Nasopharyngeal carcinoma in mice	Inhibits NPC subcutaneous tumor growth	NA	NA	[223]
		Intestinal neoplasia in mice	Inhibits RNF43 & ZNRF43 mutant intestinal epithelium	NA	NA	[224]
	IWP-2	Colorectal cancer	Suppression of Wnt ligand production	27 nM	Preclinical	[212,225]
	RXC-004	Solid tumors	Reduces tumor sizes	NA	NA	[214]
TANKs	XAV939 (XAV)	Colorectal cancer	Induces axin stabilization and inhibits colony formation of DLD-1 cells	11 nM (TNKS1), 4 nM (TNKS2)	Preclinical	[226]
		Prostate cancer	Attenuates β-catenin translocation to the nucleus	NA		[227]
		Breast cancer cells	Decreases Wnt-3a promoted cell migration in MDA-MB-231 cells	1.5 µM		[228]
		Lung adenocarcinoma	Attenuated the colony formation, proliferation, and migration of A549 cells	NA		[229]
	E7449 (2X-121)	Advanced ovarian cancer	Anti-tumor activity	50–100 nM	II	NCT03878849
		Triple-negative breast cancer	In combination with carboplatin and paclitaxel	NA	NA	NCT01618136
	AZ1366	Non–small cell lung cancer	Decreases tumor growth in combination with gefitinib	NA	Preclinical	[230]
	JW55	Colorectal cancer	Reduces Wnt signaling and tumor cell growth in SW480 cells	1.9 µM (TNKS1), 0.83 µM (TNKS2)	Preclinical	[231]
	NVP-TNKS656	Colorectal cancer	Suppresses cancer growth in APC-mutant Patient-derived xenograft models	6 nM (TNKS2)	Preclinical	[232]
	GOO7-LK	Colorectal cancer	Inhibits tumor growth in APC-mutant CRC xenograft models	46 nM (TNKS1), 25 nM (TNKS2)	Preclinical	[233]
	IWR-1	Osteosarcoma	Decreases tumor growth in combination with doxorubicin	0.18 µM	Preclinical	[212,234]
	JW74	Colorectal cancer	Downregulates Wnt target genes	790 nM	Preclinical	[235]
	TNKSi49	Colorectal cancer	Suppresses tumor growth	0.3 nM	NA	[236]
	WIKI4	Multiple cell lines	Inhibits TNKS 2 activity	15 nM (TNKS2)	NA	[237]
β-Catenin	PRI-724 (ICG-001)	Pancreatic cancer	Inhibits tumor growth	3 µM	Ib	NCT01764477
		Osteosarcoma	Attenuates cell proliferation in 143B and SJSA-1 cells	NA	Preclinical	[238]
		Acute myeloid leukemia and Chronic myeloid leukemia	Inhibits metastasis	NA	I/II	NCT01606579
		Colorectal cancer	In combination with mFOLFOX6 and bevacizumab	NA	II	NCT02413853
	CWP232228	Breast cancer stem cells	Inhibits tumor growth by attenuating β-catenin–driven transcription	0.8 µM	Preclinical	[239]
	CWP232291 (CWP 291)	Acute myeloid leukemia and chronic myeloid leukemia	Induces β-catenin degradation	273 nM	I	NCT01398462 [240]
	BC2059 (Tegavivint)	Acute myeloid leukemia	Reduces β-catenin level	NA	Preclinical	[241]
		Desmoid tumor	Primary or recurrent desmoid tumor	NA	I	NCT03459469
	LF3	Colorectal cancer	Reduces tumor growth	<2 µM	Preclinical	[242]
	MSAB	Colorectal cancer	Induces β-catenin degradation	<6 µM	Preclinical	[243]
	SAH-BCL9	Colorectal cancer	Inhibits tumor cell migration and proliferation	135 nM	Preclinical	[244]
	2,4-diamino-quinazoline	Colorectal cancer	Inhibits the β-catenin–TCF4 pathway	0.22 µM	Preclinical	[245]
	PNU-74654	Breast cancer	It enhances apoptosis and reduces β-catenin accumulation and cell proliferation. Used in combination with 5-fluorouracil	122 µM	NA	[246]
	iCRT3	Triple-negative breast cancer	Inhibits the β-catenin nuclear activity	8.2 nM	Preclinical	[247]
	PKF115-584	Colorectal cancer, Hepatocellular cancer	Inhibits tumor cell proliferation and disrupts β-catenin–Tcf complex	3.2 µM	Preclinical	[248,249]
				0.8 µM		
	PKF118-310					
	CGP049090			8.7 µM		
	AV-65	Multiple myelomas	Inhibits the growth of MM cells in the mouse model	NA	Preclinical	[250]
	CCT036477	Colorectal cancer	Inhibits tumor growth in β-catenin mutant mice	NA	Preclinical	[251]
DVL	3289-8625	Prostate cancer	Decreases tumor growth in PC-3 cells	12.5 µM	NA	[252]
	FJ9	Lung cancer and melanoma cells	Reduces tumor cell growth	Ki = 29µM	NA	[253]
Antibodies						
FZDs	Vantictumab (OMP18R5)	Breast cancer	In combination with Paclitaxel	NA	I	NCT01973309
		Pancreatic cancer	In combination with nab-paclitaxel and gemcitabine			NCT02005315
	FZD8CRD (F8CRDhFc)	Teratocarcinomas	Inhibits tumor growth		Preclinical	[254]
	OMP-54F28 (Ipafricept)	Ovarian cancer	In combination with paclitaxel and carboplatin		I	NCT02092363
		Hepatocellular cancer	In combination with sorafenib			NCT02069145
		Pancreatic cancer	In combination with nab-paclitaxel and gemcitabine			NCT02050178
	IgG-2919 (Anti-FZD5 mAb)	Pancreatic cancer	Inhibits tumor growth		Preclinical	[200]
	OTSA101 (OTSA101-DTPA-90Y)	Synovial sarcoma	Antitumor activity		I	NCT01469975 [201]
	MC-Val-Cit-PAB-MMAE	Gastric cancer			Preclinical	[255]
R-spondin3	OMP-131R10 (Rosmantuzumab)	Colorectal cancer	Inhibits tumor growth	NA	I	NCT02482441 [256]

APC, Adenomatous polyposis coli; CRC, Colorectal cancer; DVL, Dishevelled; FZDs, Frizzleds; IC50, Inhibitory concentration; IWP, Inhibitors of WNT production; Ki, Kinetic Inhibitor; NA, Not applicable; NPC, Nasopharyngeal carcinoma; PI3K Pathway, Phosphoinositide 3-kinase pathway; PORCN, Porcupine; Rnf43, RING finger protein 43; TANKs, Tankyrases; TNBC, Triple-negative breast cancer; Znrf3, zinc RING finger protein 3.

## Abbreviations

APC	Adenomatous polyposis coli
β-catenin	Cadherin-associated protein β
CSCs	Cancer stem cells
CK1	Casein kinase 1
CCND1	Cyclin D1
CKAP-4	Cytoskeleton-associated protein 4
CaMKII	Calmodulin-dependent kinase II
CAR	Chimeric antigen receptor
DCs	Dendritic cells
Dvl/Dsh	Dishevelled
DKK1	Dickkopf 1
FZD	Frizzled
GSK3β	Glycogen synthase kinase 3 β
iCRT	Inhibitors of catenin-responsive transcription
IP3	Inositol 1,4,5-triphosphate
JNK	Jun N-terminal kinase
LRP5/6	Lipoprotein receptor-related protein 5 or 6
LEF	Lymphocyte-enhancer-binding factor
MEF2	Myocyte enhancer factor 2
moAbs	Monoclonal antibodies
NFAT	Nuclear factor of activated T cells
NLK	Nemo-like kinase
NK	Natural killer cells
PCP	Planar cell polarity
PTK7	Protein tyrosine kinase 7
PI3K-AKT	Phosphatidylinositol-3 kinases-AKT
PKC	Protein kinase C
PORCN	Porcupine O-acyltransferase
RYK	Tyrosine-protein kinase
ROR1/2	Receptor tyrosine kinase-like orphan receptor 1 or 2
Rac1	Ras-related C3 botulinum toxin substrate 1
RTKs	Receptor tyrosine kinases
RSPO3	R-Spondin 3
RNF43	Ring Finger Protein 43
TAMs	Tumor associated macrophages
TCF	T-cell factor
TNKS	Tankyrase
TRF1	Telomeric repeat-binding factor 1
